# Effect of Reversal Agents on Postoperative Cognitive Disorders Following General Anesthesia in the Elderly Population: A Systematic Review and Meta-Analysis

**DOI:** 10.3390/diagnostics16040535

**Published:** 2026-02-11

**Authors:** Jing Yee Chan, Bhelvinder Singh Surinder Singh, Reshma Nachiappan, Nur Fatihah Jazlina Mohd Faizal, Zhi Xin Song, Faris Hamizan Mohd, Farah Hanim Abdullah, Azarinah Izaham

**Affiliations:** 1 Department of Anaesthesiology and Intensive Care, Faculty of Medicine, Universiti Kebangsaan Malaysia, Jalan Yaacob Latif, Bandar Tun Razak, Cheras, Kuala Lumpur 56000, Malaysia; a187738@siswa.ukm.edu.my (J.Y.C.); a190104@siswa.ukm.edu.my (B.S.S.S.); a190315@siswa.ukm.edu.my (R.N.); a181504@siswa.ukm.edu.my (N.F.J.M.F.); a187863@siswa.ukm.edu.my (Z.X.S.); a183584@siswa.ukm.edu.my (F.H.M.); farahha0301@gmail.com (F.H.A.); 2 Department of Anaesthesiology and Intensive Care, Hospital Canselor Tuanku Muhriz, Jalan Yaacob Latif, Bandar Tun Razak, Cheras, Kuala Lumpur 56000, Malaysia

**Keywords:** elderly patients, neostigmine, sugammadex, neuromuscular blockade, postoperative cognitive dysfunction

## Abstract

**Background/Objectives**: Perioperative neurocognitive disorders (PND), including postoperative delirium and cognitive dysfunction (POCD), represent significant complications in elderly surgical patients undergoing general anesthesia. The choice of neuromuscular blockade reversal agent may influence POCD risk through different mechanisms and side effects. This systematic review and meta-analysis evaluated the comparative effect of neostigmine versus sugammadex on POCD incidence in elderly patients. **Methods**: A systematic search of PubMed, Web of Science, Scopus, and Google Scholar was conducted from database inception to September 2025, following PRISMA 2020 guidelines with PROSPERO registration (CRD420251058187). Randomized controlled trials involving elderly patients (≥60 years) undergoing general anesthesia with neuromuscular blockade were included, comparing neostigmine and sugammadex for reversal. Primary outcomes included POCD incidence, assessed using validated cognitive tools, including the Mini-Mental State Examination and Montreal Cognitive Assessment. Meta-analysis was performed using Review Manager 5.4.1, with results expressed as odds ratios (ORs) and 95% confidence intervals (CIs). **Results**: Six randomized controlled trials involving 795 elderly patients published between 2017 and 2024 met the inclusion criteria. Studies encompassed non-cardiac surgery, robotic-assisted radical cystectomy, and pars plana vitrectomy. Pooled meta-analysis showed neostigmine was associated with a higher risk of POCD than sugammadex (OR 1.74, 95% CI 1.00–3.02, *p* = 0.05), with low heterogeneity (I^2^ = 39%). Secondary outcomes, including prevention of POCD, management strategies, and related complications, were inconsistently reported and unavailable across all six RCTs. Subgroup analysis stratified by neostigmine dosage demonstrated that administration of a higher dose (≥0.04 mg/kg) was associated with reduced POCD incidence compared to a lower dose (<0.04 mg/kg) (OR 0.31, 95% CI 0.15–0.63, *p* = 0.001), with negligible heterogeneity (I^2^ = 0%). **Conclusions**: This meta-analysis suggests that sugammadex may be associated with reduced early postoperative neurocognitive disorders compared to neostigmine in elderly patients, likely through rapid neuromuscular blockade reversal that minimizes residual paralysis and respiratory complications.

## 1. Introduction

Perioperative neurocognitive disorders (PNDs) represent a spectrum of cognitive impairments that occur with anesthesia and surgery in both preoperative and postoperative periods, particularly among the elderly population. Postoperative cognitive dysfunction (POCD) included cognitive decline diagnosed before operation (described as neurocognitive disorder), any form of acute event (postoperative delirium), and cognitive decline diagnosed up to 30 days after the procedure (delayed neurocognitive recovery) and up to 12 months (postoperative neurocognitive disorder), which had been associated with significant morbidity, prolonged hospital stays, increased healthcare costs and long-term functional decline [1].

Elderly patients were particularly vulnerable due to age-related neurodegenerative changes, reduced physiological reserve and the presence of comorbidities, making the effect of anesthetic and perioperative management strategies on cognitive outcomes highly relevant [2,3]. Accurate assessment of these impairments requires standardized cognitive screening tools such as the Mini-Mental State Examination (MMSE) and Montreal Cognitive Assessment (MoCA). While MMSE had long been established as a quick bedside tool, recent studies demonstrated that MoCA was increasingly preferred due to its higher sensitivity in detecting mild cognitive impairment, particularly in executive function and attention domains, which were commonly affected in POCD [4].

General anesthesia remained an essential component of modern surgical care, yet concerns persisted regarding its potential contribution to neurocognitive decline in elderly patients. Multiple factors were implicated in the pathophysiology of PND, including neuroinflammation, blood–brain barrier disruption and anesthetic-induced neurotoxicity [5]. While advances in anesthesia monitoring and perioperative care had reduced complications, cognitive impairment following surgery remained a persistent challenge in geriatric medicine.

Neuromuscular blocking agents (NMBAs) are widely used during general anesthesia to facilitate endotracheal intubation and optimize surgical conditions. At the end of surgery, reversal of neuromuscular blockade is essential to prevent residual paralysis, hypoventilation and postoperative respiratory complications. Traditionally, neostigmine, administered with an anticholinergic agent such as atropine or glycopyrrolate was the standard reversal agent. Neostigmine acts by inhibiting acetylcholinesterase, thereby increasing acetylcholine concentrations at the neuromuscular junction. However, its use is associated with muscarinic side effects including bradycardia, hypersalivation, bronchoconstriction, and increased gastrointestinal motility [6,7]. Atropine, a competitive antagonist of muscarinic acetylcholine receptors, is commonly co-administered with neostigmine to counteract these muscarinic side effects [8,9]. Unlike glycopyrrolate, atropine is lipid-soluble and readily crosses the blood–brain barrier, which may lead to central nervous system effects such as agitation, confusion or delirium, particularly in elderly patients. This property is clinically relevant in the context of perioperative neurocognitive disorders, as central anticholinergic activity may contribute to postoperative delirium or cognitive decline [10]. While atropine remains effective in mitigating peripheral cholinergic adverse effects, its potential influence on neurocognitive outcomes highlights the importance of considering alternative reversal strategies such as sugammadex.

In recent years, sugammadex has emerged as a novel reversal agent that selectively encapsulates aminosteroids neuromuscular blockers such as rocuronium and vecuronium, facilitating rapid and complete restoration of neuromuscular function independent of acetylcholinesterase inhibition. This unique mechanism distinguished it from neostigmine and spared patients from muscarinic side effects [11,12]. Numerous studies have demonstrated that sugammadex consistently achieved faster recovery times, often by several minutes, compared to neostigmine [13]. These differences in pharmacodynamics raised the question of whether the choice of reversal agent could also influence postoperative neurocognitive outcomes in elderly patients.

Several mechanisms have been hypothesized to link neuromuscular reversal agents with cognitive outcomes. Residual neuromuscular blockade (RNMB) was strongly associated with an increased risk of adverse respiratory events such as airway obstruction, aspiration, and hypoxia, which are known contributors to postoperative delirium and cognitive dysfunction. Specifically, it was found that administration of neuromuscular blockers was dose-dependently linked to a higher risk of postoperative delirium, while use of reversal agents mitigated both neuromuscular impairment and delirium risk [14]. Sugammadex enabled rapid and complete reversal of neuromuscular blockade without cholinergic activation, hence potentially reducing the incidence of perioperative pulmonary complications and hypoventilation-related stress, thereby indirectly protecting cognitive function [15].

The clinical relevance of this study was highlighted by the growing elderly surgical population worldwide. As the global population aged, the number of older adults undergoing elective and emergency procedures increased substantially. Cognitive preservation was a critical determinant of postoperative quality of life and independence in this vulnerable group. Thus, interventions that minimize the risk of PND were increasingly recognized as central to perioperative medicine and anesthetic practice.

While multiple studies have evaluated the safety and efficacy of sugammadex and neostigmine in terms of neuromuscular recovery and respiratory outcomes, relatively fewer have directly investigated their effect on neurocognitive function. Moreover, heterogeneity in study populations, variability in perioperative protocols, and differences in cognitive assessment tools such as MoCA versus MMSE have posed challenges in drawing definitive conclusions. Given these uncertainties, a systematic review is warranted to critically evaluate the available evidence on the effect of reversal agents on PND following general anesthesia in elderly patients. We hypothesize that sugammadex, due to its unique mechanism and avoidance of central anticholinergic effects, may be associated with a lower incidence of early Postoperative Cognitive Disfunctions (POCDs) up to 3 months postoperatively compared to neostigmine. This review aims to synthesize current data, identify gaps in knowledge and inform clinical practice regarding the optimal choice of reversal strategy to preserve cognitive function in this high-risk population.

## 2. Materials and Methods

### 2.1. Protocol and Registration

This systematic review was conducted in accordance with the Preferred Reporting Items for Systematic Reviews and Meta-Analyses (PRISMA) 2020 guidelines and was prospectively registered on PROSPERO (CRD420251058187). The review has been elaborated according to the PRISMA 2020 checklist in Supplementary Materials [16].

### 2.2. Eligibility Criteria

Study eligibility was determined through application of the PICOS framework, encompassing population, intervention, comparison, outcome, and study type parameters. We included studies that investigated elderly individuals (≥60 years) undergoing general anesthesia with neuromuscular blockade, in which reversal agents included either sugammadex or neostigmine. Eligible outcomes were the incidence of perioperative neurocognitive disorders, including postoperative delirium and postoperative cognitive dysfunction, assessed using validated screening tools: the Mini-Mental State Examination (MMSE) and the Montreal Cognitive Assessment (MoCA). Randomized controlled trials, observational studies, and cross-sectional studies were included. The exclusion criteria included cardiothoracic or neurosurgical patients, individuals with pre-existing neurocognitive or psychiatric disorders, those on antipsychotic medications, animal studies, reviews, and case reports. In addition, only articles published in English were considered.

### 2.3. Search Strategy

We systematically searched PubMed, Web of Science (WoS), Scopus, and Google Scholar databases from the inception of search to September 2025. Search strategies combined keywords including terms such as “adults” AND “general anesthesia” AND “reversal agents” AND “sugammadex” AND “neostigmine” AND “non-depolarising muscle relaxant” AND “postoperative neurocognitive dysfunction”.

### 2.4. Selection Process

Database search results were imported into Covidence, where duplicates were removed. The Results were then exported for title, abstract, and full-text screening. Each paper was screened by two of six independent reviewers (J.Y.C., B.S.S.S., R.N., N.F.J.M.F., Z.X.S., F.H.M.), with disagreements resolved by two second assessors (A.I. and F.H.A.).

### 2.5. Data Extraction

Data were extracted independently using a standardized form developed for the review. Extracted data items included the first authors of the article, publication year, study population, country, study design, sample size, patients’ baseline characteristics (age, surgery type, and pre-existing conditions), and outcomes, as shown in Table 1. Cognitive outcomes, measured primarily through MMSE and MoCA scores, were documented at baseline and post-surgery. Adverse effects and mortality rates were also noted.

### 2.6. Risk of Bias Assessment and Quality of Evidence

Risk of bias of the included randomized controlled trials (RCTs) was assessed using the Cochrane Risk of Bias 2.0 tool, which evaluates multiple domains including randomization procedures, allocation masking, participant and personnel masking, outcome assessor masking, outcome assessors, missing outcome data, reporting selectivity, and other sources of bias, with studies classified as having either low risk or unclear.

### 2.7. Statistical Analysis

Data analysis was performed with Review Manager (RevMan) version 5.4.1. Mean differences (MD) served as the summary measure for continuous variables, while dichotomous variables were analyzed through odds ratios (ORs); both measures included 95% confidence intervals (CI). Studies reporting medians and interquartile ranges (IQRs) underwent transformation to means and standard deviations following Cochrane Collaboration methodology via the Meta-Analysis Accelerator online platform.

Between-study variability was quantified through the Chi-squared (χ^2^) test alongside the I^2^ statistic, where values of 25%, 50%, and 75% indicated low, moderate, and substantial heterogeneity [17,18]. Analyses adhered to intention-to-treat methodology. Statistical significance was defined as a *p*-value ≤ 0.05. Forest plots provided a graphical representation of findings.

**Table 1:** Summary of extracted data.

Study ID	Study Location	Study Design	Sample Size	Age (Years)	Type of Surgery	Neuromuscular Blockers	Intervention (Reversal Agents)	Comparator	Neurocognitive Outcomes	Assessment Tools	Assessment Time Point(s)	Results
Batistaki [19]	London, England	Single-center randomized controlled trial-Parallel group	160 (from 177 recruited)	>40	Elective non-cardiac surgery	Rocuronium 0.8 mg/kg and dose when TOF > 3 twitches	Neostigmine 0.04 mg/kg IV	Sugammadex 2 mg/kg IV	Incidence of postoperative cognitive dysfunction (POCD)	MMSE	1 h postoperatively; at hospital discharge	Sugammadex group had lower incidence of POCD and faster recovery
Cao [20]	Changsha, Hunan, China	Single-center randomized controlled trial-Parallel group	196	60–85	Non-cardiac surgery under GA with cisatracurium	Cisatracurium 0.15–0.2 mg/kg TOF watch accordingly	-	Different neostigmine doses (20/40/50 μg/kg)	Incidence of postoperative cognitive impairment	MMSE	Postoperative period (within 24 h)	Optimal dose reduced residual blockade without cognitive harm
Claroni [21]	Rome, Italy	Single-center randomized controlled trial-Parallel group	109	>18	Robotic-assisted radical cystectomy	Rocuronium 0.7 mg/kg Infusion rocuronium 5 g/kg/h post-tetanic count 1&2	Sugammadex 2 mg/kg	Neostigmine 0.04 mg/kg	Neurocognitive recovery (time to orientation, awareness)	MMSE	1 h and 4 h postoperatively	Sugammadex resulted in faster recovery of orientation and extubation
Deng [22]	Dalian, China	Single-center randomized controlled trial-Parallel group	114	≥65	Non-cardiac surgery	Cisatracurium 0.15 mg/kg and Infusion of cisatracurium 0.1–0.12 mg/kg/h TOF > 2 at reversal	Neostigmine 0.04 mg/kg IV	Placebo (IV 10 mL normal saline)	Incidence of early POCD	MMSE and MoCA	Postoperative day 1	Neostigmine reduced POCD incidence compared with placebo
Kim [23]	Seoul Republic of Korea	Single-center randomized controlled trial-Parallel group	84	>60	Elective pars plana vitrectomy (PPV) with single-dose rocuronium	Rocuronium 0.6 mg/kg and TOF > 4 for reversal	Sugammadex sodium 2 mg/kg	Neostigmine methylsulfate 1 mg	Quality of recovery (Cognitive recovery domain)	MMSE	15 min postoperatively	Sugammadex showed higher QoR scores and faster recovery
Zhu [24]	Dalian, China	Single-center randomized controlled trial-Parallel group	132	65–92	Non-cardiac surgery	Cisatracurium 0.3 mg/kg and Infusion Atracurium 0.15–0.2 mg/kg/h TOF used	Neostigmine 0.02/0.04 mg/kg IV	Placebo (IV 10 mL normal saline)	Incidence of early postoperative cognitive decline	MMSE and MoCA	Postoperative day 1	Neostigmine improved cognitive function and reduced inflammation

POCD = postoperative cognitive dysfunction; MMSE = Mini-Mental State Examination; MoCA = Montreal Cognitive Assessment; GA = General Anesthesia; IV = intravenous; - = not applicable.

## 3. Results

### 3.1. Study Selection

**Figure 1:**
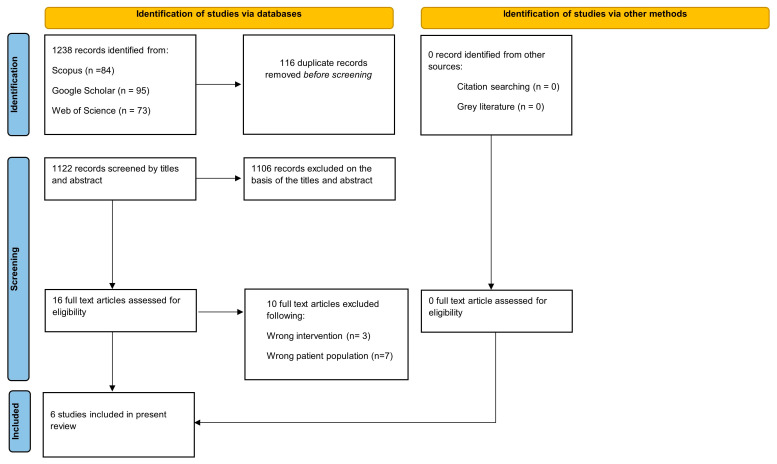
PRISMA flow diagram of literature search.

The systematic search strategy yielded 1238 records. Following deduplication, which eliminated 116 records, 1122 unique citations underwent title and abstract screening. The application of eligibility criteria resulted in the exclusion of 1106 records. Full-text evaluation was conducted for the remaining 16 articles, with 10 subsequently excluded. Six randomized controlled trials met all inclusion criteria and were incorporated into the final synthesis. Figure 1 illustrates the complete selection pathway.

### 3.2. Trial Characteristics

The data extracted from the included trials appear in Table 1. Publication dates spanned 2017 through 2024, with participant enrollment ranging from 84 to 196 individuals per study, with a total number of 795 patients. Four trials focused on patients undergoing non-cardiac surgery, and one trial each on robotic-assisted radical cystectomy and pars plana vitrectomy surgeries.

The duration of the study interventions ranged from 15 min postoperatively to the time of discharge. All included trials compared neostigmine with sugammadex, both administered intravenously. In all studies, neostigmine, sugammadex, or a placebo (normal saline) was administered at the end of surgery according to either a predetermined therapeutic protocol or the clinical judgment of the treating anesthetist, intensivist, or attending physician. Neurocognitive outcomes in all six included trials were assessed using MMSE, while only two trials additionally employed MoCA.

### 3.3. Risk of Bias Assessment

**Figure 2:**
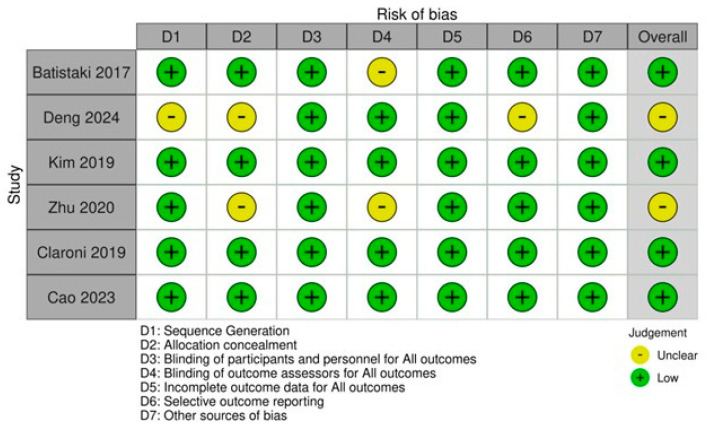
Summary of Risk of Bias [19,20,21,22,23,24].

Quality assessment of the included randomized trials employed the Cochrane Risk of Bias 2.0 instrument [25], examining seven distinct domains as presented in Figure 2. Across the six studies, most demonstrated low bias risk across all evaluated domains.

### 3.4. Outcomes

**Figure 3:**
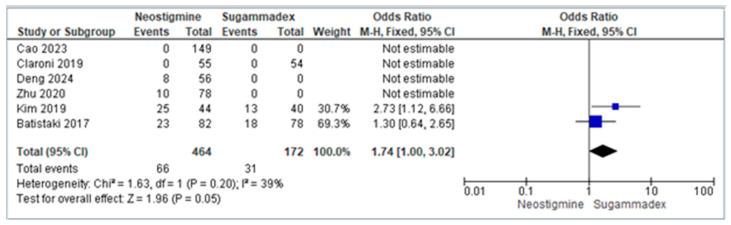
Forest plot comparing the incidence of POCD between neostigmine and sugammadex [19,20,21,22,23,24].

The pooled analysis of six randomized controlled trials compared the incidence of Postoperative Cognitive Disorders (POCD) between patients receiving neostigmine and those given sugammadex. The forest plot (Figure 3) demonstrated an overall odds ratio (OR) of 1.74, indicating that patients reversed with neostigmine had a 74% higher risk of developing POCD compared to those reversed with sugammadex. Although the confidence interval crossed unity at the lower bound, the result reached borderline statistical significance (*p* = 0.05). Importantly, the heterogeneity was low (I^2^ = 39%, *p* = 0.20), suggesting that the differences across studies were not substantial, thereby strengthening the reliability of the pooled findings.

Taken together, the meta-analysis suggests that sugammadex may provide superior protection against POCD compared to neostigmine in elderly patients undergoing general anesthesia. This effect is most likely attributable to its faster and more complete reversal of neuromuscular blockades, which reduces residual blockade, hypoxia, and related contributors to delirium and cognitive decline.

It should be noted, however, that limited information on the secondary outcomes, which encompassed prevention of POCD, treatment approaches, and associated complications, was reported in the six RCTs included in this analysis. Consequently, no substantive data can be derived regarding these outcomes.

**Figure 4:**
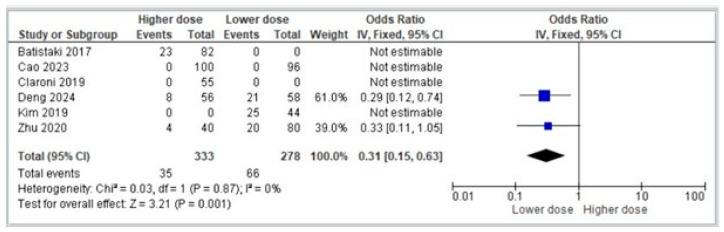
Forest plot comparing the incidence of POCD between different dosages of neostigmine [19,20,21,22,23,24].

In the subgroup analysis stratified by neostigmine dosage, studies were categorized into lower dose (<0.04 mg/kg) and higher dose (≥0.04 mg/kg) groups. Pooled data from the six randomized controlled trials demonstrated that administration of higher doses of neostigmine was associated with a significantly reduced incidence of POCD decline compared with lower doses. Specifically, the overall odds ratio favored the higher dose group (OR 0.31, 95% CI 0.15–0.63, *p* = 0.001), corresponding to an approximate 69% reduction in the odds of developing cognitive complications. Heterogeneity across the included studies was negligible (Chi^2^ = 0.03, *p* = 0.87, I^2^ = 0%), indicating consistency in the observed effect, as shown in Figure 4. While several trials reported no events in either arm [19,20,21,23] and were therefore not estimable, individual studies such as Deng et al. [22] (OR 0.29, 95% CI 0.12–0.74) and Zhu et al. [24] (OR 0.33, 95% CI 0.11–1.05) contributed to the direction of effect. These findings suggest that higher doses of neostigmine may confer a protective benefit against POCD decline compared with lower doses.

## 4. Discussion

The present systematic review and meta-analysis integrated findings from six randomized and controlled clinical studies, evaluating the effects of reversal agents, primarily sugammadex and neostigmine/atropine, on the incidence of perioperative neurocognitive disorders (PND), particularly postoperative cognitive dysfunction (POCD) in elderly patients undergoing general anesthesia. The included studies varied in population and surgical type. One study focused on robotic-assisted radical cystectomy [21], while another study focused on pars plana vitrectomy [23]. Meanwhile, the remaining four studies investigated patients who underwent non-cardiac surgery [19,20,22,24].

Deng et al. reported that neostigmine reduced POCD on the first postoperative day relative to control [22]. These findings were linked to reductions in oxidative stress markers and higher levels of brain-derived neurotrophic factor (BDNF), although no effect on inflammatory cytokines was observed. Importantly, the cognitive benefit disappeared within three to seven days, suggesting that the effect was limited to the immediate recovery phase [22]. In addition, a trial by Cao et al., which explored different neostigmine dosages (20–50 μg/kg), confirmed faster neuromuscular recovery but reported no differences in postoperative cognitive outcomes [20]. This implies that improved train-of-four ratios do not necessarily translate into neurocognitive protection [20].

Sugammadex provides rapid and predictable reversal of aminosteroid neuromuscular blockers without cholinergic side effects. In comparative studies, patients treated with sugammadex exhibited early physiological recovery and, in certain cases, immediate postoperative cognition. For example, Kim et al. reported higher recall ability at 15 min after surgery [23], while Claroni et al. documented higher MMSE scores at one and four hours [21]. These improvements, however, did not persist beyond the early recovery period. Furthermore, Batistaki et al. reported no significant differences in the incidence of POCD between sugammadex and neostigmine at either one hour or at hospital discharge [19]. Nevertheless, through the studies, sugammadex achieved faster and more reliable reversal, highlighting its pharmacological superiority.

The overall evidence indicates that both agents influence early postoperative recovery, but neither consistently prevents POCD beyond the immediate postoperative period. Comparisons showed that sugammadex enabled faster physiological recovery and occasionally better short-term cognitive scores. However, Batistaki et al., being the largest trial among the six included RCTs, reported no clinically significant differences in POCD incidence between sugammadex and neostigmine [19]. Conversely, neostigmine appeared to reduce early POCD in elderly patients compared with placebo, though the benefit was transient.

Based on the pharmacokinetic profiles of neostigmine and sugammadex, they would be expected to result in less POCD in elderly patients through several key mechanisms. Neostigmine, an acetylcholinesterase inhibitor, increases acetylcholine non-selectively in both the peripheral and central nervous systems, potentially interfering with cognitive function [6]. Critically, neostigmine requires co-administration with anticholinergic agents such as atropine, which is lipid-soluble and crosses the blood–brain barrier, causing central nervous system effects including confusion and delirium, which are particularly problematic in elderly patients [8]. In contrast, sugammadex acts through selective encapsulation of rocuronium and vecuronium without any cholinergic receptor interaction or central nervous system penetration, avoiding both cholinergic disruption and anticholinergic burden [11].

Additionally, sugammadex provides rapid and complete neuromuscular blockade reversal, preventing residual neuromuscular blockade and associated complications such as hypoxemia and hypercarbia, which are known contributors to cognitive dysfunction [12]. We also demonstrated that neostigmine was associated with 74% higher odds of developing POCD compared to sugammadex (OR 1.74, 95% CI 1.00–3.02, *p* = 0.05), with individual trials showing higher Mini-Mental State Examination scores and better early cognitive recovery in sugammadex-treated patients. This cleaner pharmacological profile positions sugammadex as superior for preserving early postoperative cognitive function in elderly surgical populations.

The six included RCTs provide useful insights into the potential cognitive effects of neuromuscular blockade reversal agents, though several limitations must be acknowledged. At the study level, most trials enrolled relatively small cohorts, with only two enrolling more than 150 participants. Methods of cognitive assessment varied considerably (MMSE, MoCA, Postoperative Quality Recovery Scale, Isaacs Set Test), which complicates direct comparison. Furthermore, follow-up was often limited to hours or a few days, whereas POCD may develop weeks after surgery. Other than that, POCD may be influenced by multiple confounding factors, including patient-related factors, e.g., advanced age, frailty, multimorbidity, pre-existing neurocognitive impairment, impaired mobility, malnutrition, and polypharmacy; surgery-related factors, including prolonged or extensive procedures with significant tissue trauma and inflammation; and anesthesia-related factors, e.g., depth and duration of anesthesia [26].

At the review level, the modest number of eligible studies (n = 6) restricted the overall generalizability of findings. Substantial heterogeneity in outcome definitions and assessment time points further limited the strength of conclusions that could be drawn. Nevertheless, by bringing together trials published between 2017 and 2024, this review reflects contemporary anesthetic practice and highlights key evidence gaps that warrant further research.

Across the included randomized controlled trials, neurocognitive outcomes were assessed at markedly different postoperative intervals, ranging from as early as 15 min and 1 to 4 h after anesthesia emergence to day 1 postoperatively and at hospital discharge. Given the time-dependent and fluctuating nature of perioperative neurocognitive disorders, pooling outcomes measured at different time points may reduce internal validity by combining transient emergence-related cognitive effects with more sustained postoperative cognitive dysfunction [27,28]. Heterogeneity in outcome assessment time points represents an important limitation of this meta-analysis. Although statistical heterogeneity was low, methodological variability in assessment timing warrants cautious interpretation of the findings and highlights the need for future trials with standardized cognitive assessment intervals [18].

These findings have important clinical implications. Although preliminary evidence suggests that sugammadex may be associated with a lower risk of early POCD compared with neostigmine, current findings are insufficient to shape definitive clinical recommendations. For clinical practice, the choice of reversal agent should not be based solely on expectations of cognitive benefit.

Neostigmine remains a cost-effective and widely available option that may confer very short-lived cognitive advantages [29]. Sugammadex, by contrast, provides rapid and predictable reversal, which may be particularly valuable in high-risk patients who require early extubation or prompt neurological evaluation [30], but its higher cost must be justified by factors other than POCD.

Ultimately, cognitive outcomes after surgery are determined by a complex interplay of patient comorbidities, surgical invasiveness, anesthetic management, and perioperative complications [28]. The reversal agent is only one factor in this broader context, and anesthetists should individualize the choice of reversal drug according to patient characteristics, surgical context, and drug availability until higher-quality evidence is available.

Future research should aim to overcome the current limitations by conducting large, multicenter randomized controlled trials with adequate power to detect differences in the incidence of POCD. The use of standardized and validated neurocognitive assessments, combined with longer follow-up periods extending from weeks to months, will be essential to capture both short- and long-term outcomes. Mechanistic studies are also needed to clarify how reversal agents influence cerebral physiology, oxidative stress pathways, and neurotrophic factors such as BDNF, which may provide valuable insight into potential protective effects.

A key strength of this review lies in its contemporary scope, bringing together the most recent trials to highlight current evidence and knowledge gaps. By synthesizing findings across diverse surgical populations, this study highlights that while sugammadex and neostigmine play critical roles in safe neuromuscular reversal, their influence on neurocognitive outcomes remains limited and uncertain.

## 5. Conclusions

This systematic review and meta-analysis suggest that sugammadex may be associated with a lower incidence of early postoperative cognitive dysfunction (POCD), compared with neostigmine in elderly patients undergoing general anesthesia. Its potential advantage is likely linked to rapid and complete reversal of neuromuscular blockade, thereby reducing residual paralysis, hypoxia, and other secondary contributors to cognitive impairment. Nonetheless, the largest available trial demonstrated no clinically significant difference in POCD incidence between these two agents. The selection of a reversal agent should therefore be guided primarily by pharmacological efficacy, patient safety, and resource availability, rather than expectations of cognitive benefit.

## Data Availability

The data are contained within the article.

## Supplementary Materials

The following supporting information can be downloaded at https://www.mdpi.com/article/10.3390/diagnostics16040535/s1, Supplementary File S1: PRISMA 2020 checklists are cited in the Supplementary materials.

## Abbreviations

The following abbreviations are used in this manuscript:

CI	Confidence intervals
IQRs	Interquartile ranges
OR	Odds ratios
POCD	Postoperative cognitive dysfunction
PND	Perioperative neurocognitive disorders
MMSE	Mini-Mental State Examination
MD	Mean differences
MoCA	Montreal Cognitive Assessment
NMBAs	Neuromuscular blocking agents
RCTs	Randomized controlled trials
RNMB	Residual neuromuscular blockade
PRISMA	Preferred Reporting Items for Systematic Reviews and Meta-Analyses
χ^2^	Chi-squared
